# Mitogen-activated protein kinase kinase 7 in inflammatory, cancer, and neurological diseases

**DOI:** 10.3389/fcell.2022.979673

**Published:** 2022-10-20

**Authors:** Amada D. Caliz, Anastassiia Vertii, Vijay Fisch, Soonsang Yoon, Hyung-Jin Yoo, John F. Keaney, Shashi Kant

**Affiliations:** ^1^ Division of Cardiovascular Medicine, Department of Medicine, Brigham and Women’s Hospital, Harvard Medical School, Boston, MA, United States; ^2^ Department of Molecular, Cell and Cancer Biology, University of Massachusetts Chan Medical School, Worcester, MA, United States

**Keywords:** signaling, stress, MAPKs, senescence, inflammation, cancer, neuron

## Abstract

Stress-activated mitogen-activated protein kinase kinase 7 (MKK7) is a member of the dual-specificity mitogen-activated protein kinase family. In the human body, MKK7 controls essential physiological processes, including but not limited to proliferation and differentiation in multiple tissues and organs. MKK7, along with the MKK4 pathway, has been implicated in stress-activated activities and biological events that are mediated by c-Jun N-terminal kinase (JNK) signaling. Although numerous studies have been performed to identify the role of JNK in multiple biological processes, there are limited publications that focus on dissecting the independent role of MKK7. Recent research findings have spurred testing *via in vivo* genetically deficient models, uncovering previously undocumented JNK—independent functions of MKK7. Here we discuss both JNK—dependent and—independent functions of MKK7 *in vivo*. This review summarizes the role of MKK7 in inflammation, cytokine production, cancer, and neurological diseases.

## Introduction

**FIGURE 1:**
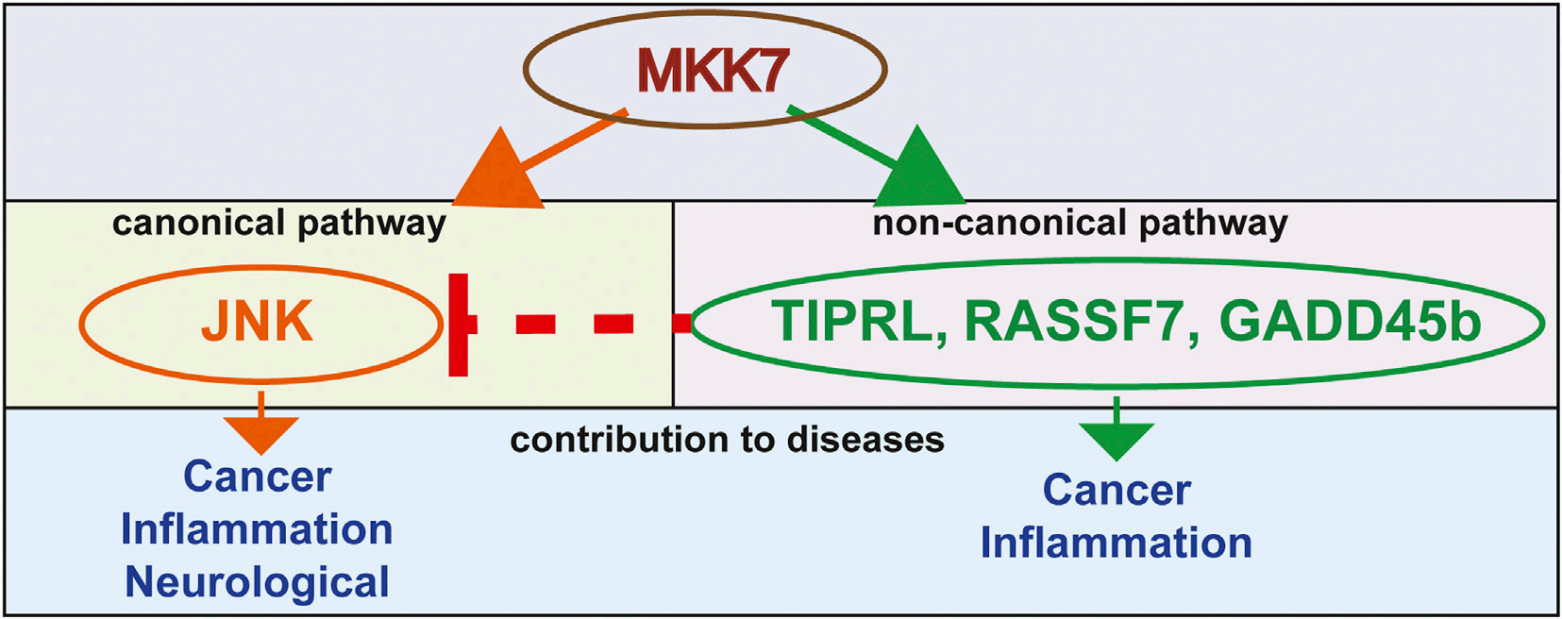
A model of MKK7-mediated mechanisms that contribute to a variety of diseases.

Mammalian mitogen-activated protein kinases (MAPKs) ERK1/2 were discovered and cloned in the late ‘80s and early ’90s (Sturgill et al., 1988; Boulton et al., 1990; Robinson and Cobb 1997; Avruch 2007). Later, based on sequence similarity with ERK1/2, different members of MAPKs were identified, including JNK and p38 MAPKs (Derijard et al., 1994; Han et al., 1994; Rouse et al., 1994). Currently, the MAPK signaling network consists of six different pathways: ERK1/2, JNK1/2/3, p38α/β/γ/δ, ERK3/4, ERK5, and ERK7/8 (Olea-Flores et al., 2019). Two major pathways, the c-Jun N-terminal kinase (JNK) and the p38 MAP kinase (p38), form the stress-activated protein kinase family (SAPK) (Davis 2000). There are several stress stimuli responsible for the activation of JNK and p38 MAPK, including ultraviolet (UV) radiation, cytokines, free fatty acid (FFA), and reactive oxygen species (ROS), to name a few (Davis 2000; Craige et al., 2019). The response of these pathways illustrates MAPKs’ ability to control a range of critical cellular functions, such as cytokine production, immune response, apoptosis, differentiation, stress response, cell survival, and cell proliferation (Cargnello and Roux 2011). The activation of stress-activated MAPK, JNK, and p38 are mediated by various members of the evolutionary preserved upstream mitogen-activated protein kinase kinase (MAP2K) family members (Davis 2000; Wang et al., 2007; Cargnello and Roux 2011), which includes mitogen kinase kinase 3 (MKK3), mitogen kinase kinase 4 (MKK4), mitogen kinase kinase 6 (MKK6) and mitogen kinase kinase 7 (MKK7). Such variety in the mammalian repertoire of upstream activators might, in turn, explain the specificity of the stimuli-induced pathway activation. The central dogma defines MKK4/7 as major activators of the JNK pathway (Figure 1) and MKK3/6 as responsible for the activation of p38 MAPKs (Davis 2000; Cargnello and Roux 2011). However, recent studies have clearly demonstrated that there are overlaps of MKKs functions in the regulation of JNK and p38 MAPK *in vivo* (Caliz et al., 2021)*. In vitro*, JNKs are activated *via* the sequential phosphorylation of upstream protein kinases that comprises two dual-specificity MAP kinase kinases (MKK4 and MKK7) [4]. Stress stimuli such as cytokines, UV, and FFA first activate mitogen-activated protein kinase kinase kinase (MAP3Ks) family members. After activation of MAP3Ks family members, the upstream activators of MAP3Ks phosphorylate and activate MKK4 and MKK7, which, in turn, activates JNK by dual phosphorylation of both threonine and tyrosine residues within a Thr–Pro–Tyr motif in the protein kinase subdomain VIII. Both MKK4 and MKK7 belong to the MAP2K family members. While both MKK4 and MKK7 have been implicated in the activation of JNK, MKK4 also can phosphorylate the Thr–Gly–Tyr motif of p38 MAPKs (Davis 2000; Wang et al., 2007; Cargnello and Roux 2011; Craige et al., 2016).

The first member of the stress-activated MAP2K (MKK) family was cloned and identified as XMEK2 in *Xenopus laevis* (Yashar et al., 1993). Eventually, mammalian homologs of XMEK2 were cloned in mammals. First, stress-activated MAP2K cloned in mice and humans were named SEK1 [for stress-activated protein kinase (SAPK)/extracellular-signal-regulated protein kinase (ERK) kinase-1]. SEK1 was later called MKK4 or JNKK1 (JNK kinase 1) (Sanchez et al., 1994; Derijard et al., 1995; Lin et al., 1995).

Subsequently, the other member of the MKK family was cloned by a different laboratory group and they called it SEK2, JNKK2, or MKK7 (Lawler et al., 1997; Tournier et al., 1997; Wu et al., 1997; Yao et al., 1997). Mammalian MKK7 and MKK4 proteins reveal 55% similarity to each other within their kinase domains. MKK7 proteins are encoded by the MAP2K7 gene in humans. MAP2K7 gene is made of 12 exons and presents on chromosome location 19p13.2. In comparison, the MAP2K7 gene in mice composes of 14 exons and is on chromosome location 8A1.1. Additionally, the MAP2K7 gene can undergo alternative splicing to generate six different isoforms of MKK7 in mice (Tournier et al., 1999). These six isoforms of MKK7 are made of a collection of protein kinases because of their differences at N-termini (*α*, *β*, and *γ* isoforms) and C-termini (1 and 2 isoforms) (Tournier et al., 1999). These isoforms of MKK7 vary in length and comprise 345 to 467 amino acids. MKK7 isoforms contain a molecular mass between 38 and 52 kDa. Nevertheless, the functional importance of different MKK7 isoforms is still not known. A comparison of different MKK7 isoforms as an activator of JNK demonstrates that a short isoform of MKK7, MKK7α, lacks the NH2-terminal extension and displays a lower basal activity compared to MKK7β and γ isoforms (Tournier et al., 1999). This difference in the ability to activate JNK kinase can be explained by the capability of the longer isoforms MKK7β and γ to bind JNK *via* three-D domains which is a cluster of positively charged amino acids surrounded by hydrophobic amino acids and present in the N-terminus isoforms MKK7β and γ but not in MKK7α (Tanoue and Nishida 2003; Ho et al., 2006). Therefore, MKK7β and γ isoforms can bind to the C-terminal kinase lobe of the MAPKs known as the common docking (CD) domain *via* their D-domain (Tanoue and Nishida 2003). MKK7 gene orthologs exist in different organisms, including mammals, reptiles, amphibians, and zebrafish. The first genetic evidence of the MKK7 critical role in development came to light as early embryonic death was triggered by the targeted deletion of the MKK7 genes in mice *in vivo* (Yang et al., 1997a; Nishina et al., 1997; Ganiatsas et al., 1998). The MKK7^−/−^ embryos die due to anemia and abnormal hepatogenesis between E11.5 to E13.5 (Ganiatsas et al., 1998; Nishina et al., 1999; Watanabe et al., 2002; Wada et al., 2004).

Stress-activated MAPK pathways comprise mainly a three-tier system in which MAP2K, like MKK4 and MKK7, exhibit phosphorylation on their Ser and Thr residues of the S-X-A-K-T motifs in the Kinase domain by their upstream activator of mitogen-activated protein kinase kinase kinase (MAP3K) family members (Davis 2000; Asaoka and Nishina 2010). MAP3Ks encompass several family members in the mammalian system, which include Mitogen-activated protein kinase kinase kinase 1–4 (MEKK1-4), Transforming growth factor-β-activated kinase 1 (TAK1), Mixed-lineage kinase 1–4 (MLK1-4), Apoptosis signal-regulating kinase 1–3 (ASK1-3), Thousand and one amino acid (TAO) kinases 1–3 (TAOK1-3), Tumor progression locus 2 (TPL2), Dual leucine-zipper bearing kinase (DLK), Leucine-zipper kinase (LZK), and Zipper sterile-alpha motif kinase (ZAK) (Qi and Elion 2005; Morrison 2012; Craige et al., 2016; Craige et al., 2019). Once MKK7 binds to its upstream regulators of MAP3Ks, causing activation, the subsequent downstream substrate JNK is activated by phosphorylation on the JNK dual phosphorylation site of Threonine and Tyrosine residues by MKK7.

The activation of the MAPK stress pathway requires several protein components to be in the same place. Therefore, scaffold proteins play an important role in bringing these signaling cascade members to the same place at the same time when stimuli are present. The interactions of the scaffold proteins binding with JNK-interacting protein 1–4 (JIP1-4) and Plenty of SH3 (POSH) demonstrate the critical role in which stress stimuli activate the MKK7 pathway, and its downstream components play in performing their function (Whitmarsh et al., 1998; Whitmarsh et al., 2001; Xu et al., 2003; Whitmarsh 2006). Additionally, the phosphorylation of scaffold proteins is sometimes required for the full function of this pathway. Thus, MAP3Ks family members have been known to facilitate this interaction by phosphorylating the scaffold proteins (D’Ambrosio et al., 2006; Kant et al., 2017), which might be changing their conformations. Recent studies have shown that scaffold proteins, such as JIP and POSH, play a dynamic role during the activation of stress-activated MAPKs. Other than JIPs and POSH, MKK7 also binds to the Receptor for activated C kinase 1 (RACK1) (Guo et al., 2013). The interaction of RACK1 with MKK7 facilitates the association of MKK7 and MAP3Ks, thereby enhancing MKK7/JNK activity. Recent studies have also shown that FFA-activated JNK requires the intact JIP1 and MKK7 for the activation and function of this pathway *in vitro* and *in vivo* (Kant et al., 2017).

## Non-canonical pathway

JNK is the primary downstream target of MKK7 and MKK4 (Wang et al., 2007). The canonical MKK7 pathway has been known for a while and mainly uses JNK downstream to activate its biological responses (Tournier et al., 2001; Park et al., 2019). Nonetheless, some studies demonstrate that MKK7 functions by other downstream proteins and regulates biological processes in a non-canonical way. One recent study has shown that MKK7 interacts with the Ras-association domain family 7 (RASSF7) (Takahashi et al., 2011). This interaction suppresses the UV induced MKK7 interactions and phosphorylation of JNK, which leads to the anti-apoptotic outcome, DNA mutation and cancer (Takahashi et al., 2011). Other examples of MKK7 non-canonical pathways involve its binding to Growth arrest and DNA damage-inducible beta (GADD45b) (Papa et al., 2004; Rega et al., 2018) and TOR signaling pathway regulator-like (TIPRL) (Yoon et al., 2017) proteins. The GADD45-beta and TIPRL involved non-canonical pathways of MKK7 and an effector molecule. GADD45-beta directly binds to MKK7 and blocks its catalytic activity, suppressing the MKK7-JNK pathway. This binding of GADD45-beta to MKK7 is required to antagonize tumor necrosis factor-alpha (TNFα) -induced cytotoxicity in the cells. If a peptide inhibiting this binding of GADD45-beta to MKK7 has been used, then TNFα -induced cytotoxicity can be restored. TIPRL inhibits MKK7-JNK signaling by mediating the interaction between MKK7 and Protein phosphatase type 2A catalytic subunit protein (PP2Ac). This interaction between MKK7 and PP2Ac also restricts the prolonged activation of MKK7, which leads to the prevention of apoptosis (Yoon et al., 2017) (Figure 1).

Studies have also displayed the JNK-independent role of MKK4 (Hickson et al., 2006; Caliz et al., 2021). Previously, MKK4 has been shown to interact with p38 MAPKs (Brancho et al., 2003; Wang et al., 2007). However, a recent study has shown that MKK7 can also affect p38 signaling *in vivo* (Caliz et al., 2021). This non-canonical role of MKK7 has not been adequately explored. Therefore, further research is necessary to determine the role of MKK7 in its new non-canonical pathway.

## Inflammation and cytokine production

As the initial line of defense against harmful pathogens, the innate immune system is an evolutionarily conserved response (Smith et al., 2019). Pathogens, such as bacteria, initiate the orchestration of the synchronous event. The subsequent production of inflammatory cytokines is caused by the quick activation of the MAPK signaling pathway mediated by this response. This MAPK-activated inflammatory cytokine production further drives the amplification of the inflammatory response affecting both the immune and non-immune cells in our body (Canovas and Nebreda 2021). Chronic inflammation follows when the acute inflammatory mechanism is not able to eliminate tissue injury and inflammatory cytokines in time (Lintermans et al., 2014). The inflammatory response must be suppressed to prevent progression from acute to chronic inflammation to avoid tissue damage. Therefore, deciphering the responsible mechanisms, both cell-type- and stimuli-specific, are vital to regulating uncontrolled inflammatory responses and cytokine production (Chen et al., 2018). The specific manner in which MKK7 contributes to the macrophage-specific immune response has not been identified. However, a recent article described the use of both *in vivo* and *in vitro* models to determine MKK7 kinase contribution with respect to downstream target activation, cytokine secretion, macrophage migration, and macrophage polarization (the essential characteristics of an effective immune response) (Caliz et al., 2021). Pro-inflammatory cytokines, such as tumor necrosis factor-alpha (TNFα) and bacterial lipopolysaccharides (LPS), activate stress-activated MAPKs family members, JNK, and p38 (Kant et al., 2011). Although, it has been established that another group of MAP2K, MKK3/6, play an important role in activation of p38 signaling, it is unclear which MAP2K plays a more prominent role during TNFα and LPS activation of JNK and p38 in macrophages. Studies have shown the major role of MKK7 in TNFα and LPS-activated JNK signaling (Tournier et al., 2001; Caliz et al., 2021). But the function of MKK7 in p38 activation in macrophages has remained unknown.

A recent study may possibly be the first evidence that it has shown a role of MKK7 in p38 MAPK activation in macrophages. Specifically, this study concludes that MKK7 is required for full activation of p38 MAPK (Caliz et al., 2021). Additionally, various studies have clearly demonstrated that LPS-mediated cytokine production of TNFα, IL1α, IL1b, and IL6 requires MKK7 presence in macrophages *in vitro* and *in vivo* (Tournier et al., 2001; Caliz et al., 2021). Additionally, the LPS-mediated inflammatory response in macrophages was shown to be contributed by MKK4. Although in comparison to MKK7, MKK4’s effects on JNK signaling and cytokine production were modest (Caliz et al., 2021) (Table 1). For healthy macrophages to execute their proper function, robust migration and invasion are required. Notably, significant blockage formed in the migration and invasion of macrophages in MKK7-deficient mice compared to the control. Similar to the migration and invasion data, MKK7 is essential for macrophage polarization to M1 inflammatory macrophage and, therefore, for inflammatory cytokine production (Caliz et al., 2021). The ability of MKK7 to control polarization and migration of macrophages suggests that MKK7 contributes to the regulation of inflammatory responses (Caliz et al., 2021). Data from this study illustrate the MKK7 pathway as a plausible target for therapeutic drug development beneficial for the treatment of sepsis and inflammation.

**TABLE 1:** MKK7 deficiency -associated defects in mammals.

Tissue/Disease models	MKK7 deficiency -associated defects	References
Macrophage	Defect in migration, invasion, cytokine production	Caliz et al. (2021)
Prostate cancer	Rapid development of tumor	Hubner et al. (2012); Atala (2013)
Lung carcinoma	Less survival and more tumor	Schramek et al. (2011)
Mammary tumor	Loss of tumor suppression	Schramek et al. (2011)
Neuron during development	Defect in axon elongation	Yamasaki et al. (2011), Yamasaki et al. (2017), Shin et al. (2021)
Stress to the retinal ganglion cells	More survival	Syc-Mazurek et al. (2018)
Neuron during aging	Motor dysfunction and axonal degeneration	Yamasaki et al. (2011), Yamasaki et al. (2017)
Behavioral response	Depression	Shin et al. (2021)

Rheumatoid arthritis (RA) affects almost all joints in our body which includes hands and knees. RA is one of the most common inflammatory diseases. RA is characterized by synovial inflammation and joint destruction (Firestein 2003). Uncontrollable cytokine production and extracellular matrix degradation by matrix metalloproteinase (MMP) play an important role during RA development. Previously, a report suggested that MKK7 inhibition using antisense oligonucleotides (ASO) decreases the severity of disease in K/BxN serum transfer arthritis (STA) mice model of Rheumatoid arthritis (Lee et al., 2012). These studies have shown that MKK7 not only controls cytokine production during inflammation but also regulates extracellular matrix degradation by matrix metalloproteinase (MMP), in which both can have synergistic effects on inflammatory diseases like Rheumatoid arthritis.

## Cancer

Chronic inflammation has been associated with cancer development (Coussens and Werb 2002). On the other hand, senescence can suppress tumor development (Zeng et al., 2018). A balance between proliferation, differentiation, senescence, and apoptosis is required for tissue homeostasis. Cancer cells usually escape by altering this balance and homeostasis in tissues. MKK7 has been implicated for its role in the process of programming cell death. For example, data from a recent publication suggest the role of MKK7 in apoptosis. In this study, authors have shown the apparent contribution of MKK7 during viral-activated mitochondrial antiviral signaling protein (MAVS/VISA/Cardif/IPS-1) induced apoptosis (Huang et al., 2014).

Cancer cells usually escape apoptotic signaling and favor proliferation. Previously, the stress kinase MKK7 has been coupled with p53 stability and tumor suppression. Moreover, the inactivation of MKK7 has been shown to induce tumor growth. Specifically, the authors have demonstrated that MKK7 is required for tumor suppression and overall survival using two different tumor models: KRasG12D-driven lung carcinomas and HER2/Neu (NeuT) oncogene-driven mammary tumors. This process of MKK7-mediated tumor suppression requires JNK1/2 and p53 protein intact, as a deficiency in either JNK1/2 or p53 can yield in tumor progression (Schramek et al., 2011).

Additionally, two independent studies that utilized the prostate cancer model in MKK7 knockout mice with MKK4 and Phosphatase and tensin homolog (PTEN) deficiency showed rapid development of invasive adenocarcinoma (Hubner et al., 2012; Atala 2013). PTEN is a phosphatase family member (He et al., 2021). Mutations of this phosphatase gene are known in the development of many cancers, including prostate cancer (Li et al., 1997; He et al., 2021). On the other hand, MKK4, a kinase that works oppositive to phosphatases, has been found to have a loss of function mutation in 5% of human cancer (Whitmarsh and Davis 2007) (Table 1).

On the contrary, some reports show that MKK7 is required for liver metastasis of colon cancer cells (Sakai et al., 2014). miR-493 works as a suppressor of liver metastasis in part by MKK7 inhibition. This difference in the MKK7 role may be explained by the context and cell type-specific role of MKK7, which results in a difference in the function of its downstream targets. It needs to be examined further as MKK7 might play different roles in tumor development and metastasis. Also, it is possible that distinct MKK7 isoforms offset different roles during tumor progression. 

Aneuploidy is one of the hallmarks of cancer cells and often derives from supernumerary centrosomes that form multipolar spindles, resulting in unequal separation of the chromosomes into daughter cells during mitosis. Therefore, centrosome duplication is an essential process, well regulated by a Polo-like kinase 4 (PLK4)—the unscheduled activity of PLK4 results in centrosome overduplication. Intriguingly, stress-activated MAPKs such as MKK7 and MKK4 prevent centrosome overduplication, thus protecting cells from aneuploidy (Nakamura et al., 2013).

Another study conducted on lung cancer patients examined five different polymorphisms of MKK7 (p.Glu116Lys, p.Asn118Ser, p.Arg138Cys, p.Ala195Thr, and p.Leu259Phe). Results showed that out of all five mutations, patients with one specific mutation, MKK7 p.Glu116Lys polymorphism, displayed lung cancer metastasis at a significantly higher rate than all other mutations (Table 1) (Qiu et al., 2016). The above study clearly showed a link between MKK7 mutations and human cancers (Qiu et al., 2016). These studies suggest that strengthening our understanding of the function of MKK7 proteins may allow the development of potential cancer therapies in the future (Park et al., 2019).

Overall, there is a complex relationship between MKK7 and cancer progression: while MKK7 promotes apoptosis, suppresses tumor growth, and protects cells from aneuploidy, MKK7 is required for metastasis and inflammation.

## Neurological diseases

MKK7 expresses abundantly in neurons. Recently, several studies have suggested its role in neuronal function, specifically during neurite development, axonal degeneration, and neuronal survival. For example, an MKK7 mRNA localizes to the cone of a neurite with the potential to be translated. Translated MKK7 protein is then phosphorylated within the neurite shaft and acts together with JNK1 and dual leucine zipper kinase (DLK) to phosphorylate Map1b, a protein responsible for microtubule bundling, thus leading to elongation of neurites (Feltrin et al., 2012). Notably, the mechanism of engagement of activated JNK1- MKK7 signaling module in the neurite shaft also prevents JNK1 nuclear translocation and transcriptional activities.

The first study suggesting MKK7 plays a role in the development of neurons utilized Nestin promoter-driven Cre recombinase (Nestin-Cre) to generate a neuronal tissue-specific deletion of MKK7 knockout. Though the phenotype of mutant mice presented no distinguishable differences in their appearance from their control littermates during embryogenesis, neonates died immediately after birth. Histological examination confirmed severe brain development deficiencies, including enlarged ventricles, minimal axon tracts, and reduced striatum (Table 1). Moreover, an anomalous accumulation of filamentous structures and autophagic vacuoles in *the MKK7* knockout brain was revealed *via* electron microscopy (Yamasaki et al., 2011). This study clearly showed that MKK7 plays a role in axon elongation.

Another study showed that Mkk7 deficiency reduced JNK signaling in retinal ganglion cells (RGCs) after axonal injury, which led to a significantly greater percentage of surviving RGCs 35 days after controlled optic nerve crush (CONC) as compared to wild-type controls (Mkk7: 29.1%, WT: 15.2%; *p* < 0.001) (Syc-Mazurek et al., 2018), indicating the critical role of MKK7 in axon injury.

A recent study demonstrated that neuron-specific MKK7 knockout mice exhibited abnormal circadian rhythmic behaviors as well as decreased locomotor activity. Eight months old neuron-specific MKK7 knockout mice showed motor dysfunctions, such as weakness of hind-limb and gait abnormality in this study, suggesting a possible age-dependent variable. Additionally, muscle atrophy and axonal degeneration in the spinal cord were also observed in these aged neuron-specific MKK7 knockout mice (Table 1) (Yamasaki et al., 2017).

A recent article showed the role of MKK7 with MKK4 in the impairment of hippocampus neurons. Specifically, the deletion of Mkk4/Mkk7 induced a misalignment position of immature hippocampal neurons and alterations in their dendritic architecture pattern and maturation process (Castro-Torres0 et al., 2021). Of particular interest is work where the authors examined the role of parental behavior in MKK7 knockout mice. This study used a different neuronal tissue-specific promoter-driven Cre recombinase, Synapsin I (Syn-Cre), to delete MKK7 only in mature neurons.

These data revealed that in comparison to the controls, MKK7 knockout mice presented normal locomotor functions and cognitive ability, although depression allied behavior was also exhibited. In addition to the depression allied behavior, mRNA expression of genes related to the calcium channel as well as the neural signaling pathways were decreased. This study suggested the potentially important role in which MKK7 is responsible for regulating gene expression related to behavioral patterns such as promoting normal social behavior or depression (Table 1 (Shin et al., 2021).

Excitotoxicity following cerebral ischemia leads to neuronal death, and the MKK-JNK pathway has a key role in excitotoxic cell death. For example, the inhibition of MKK7 signaling using a peptide inhibitor of GADD45beta showed reduced neuronal death in neurotoxicity *in vitro* and neuroprotection in two models of brain ischemia (Vercelli et al., 2015). Also, MKK7 or MKK4 deletion in retinal ganglion cells increased cell survival upon optic injury. Notably, Jip1 (scaffolding protein of MKK and JNK) deletion also showed increased ganglion cell survival by inhibiting proapoptotic MKK-JNK signaling. It will be interesting to determine whether temporary inhibition of MKK4 or MKK7 signaling could increase ganglion cell survival upon cell transplantation. Thus, MKK7 contributes to neuronal functions during development and adulthood, making MKK7 an essential candidate for brain health.

## Concluding remarks

Recent studies have implicated the role of the MKK7 signal transduction pathway in many pathological conditions, including inflammation, cancer, and neurological diseases. The resulting cytokine signaling mediated by the MKK7 pathway leads to the downstream stress kinase JNK and p38 MAPK activation, which leads to the transcriptional activation of cytokine-producing genes and, ultimately, cytokine production (Davis 2000; Tournier et al., 2001; Sabio and Davis 2014; Craige et al., 2016; Caliz et al., 2021). Biochemical studies using cultured primary cells and various mice models have provided substantial evidence to support this conclusion (Tournier et al., 2001; Wang et al., 2007; Caliz et al., 2021). MKK7 has also been implicated in several neurodegenerative diseases (Yang et al., 1997b). MKK7 expresses relatively high in neurons and is required for many neurological functions. The MKK7 signaling pathway, therefore, represents a potential target for therapeutic intervention. Future studies will aim to establish whether MKK7 directly contributes to these disease processes or if MKK4 is required for fully functional downstream activation.

We have yet to solve a fundamental question about the competency of cell signaling when interpreting MKK7 activation (e.g., survival signaling versus apoptosis). Though emerging data supports both MKK7 and MKK4 functioning independently of JNK, further research is needed to understand its essential role in these non-canonical pathways fully (Figure 1).

In conclusion, progress has been made toward understanding the *in vivo* function of the MKK7 pathway. However, further studies are necessary to dissect the cell type-specific mechanisms and determine its temporal role in combination with the other components of the stress signaling pathway.
